# Role of open nephron sparing surgery in the era of minimal invasive surgery

**DOI:** 10.4103/0970-1591.57930

**Published:** 2009

**Authors:** Gaurav Gupta, Sameer Grover, Santosh Kumar, Nitin S. Kekre

**Affiliations:** Department of Urology, Christian Medical College and Hospital, Vellore - 632004, Tamilnadu, India

**Keywords:** Renal cell carcinoma, nephron sparing surgery, partial Nephrectomy, open, laparoscopic partial nephrectomy

## Abstract

**Objective:**

The study aims to review the current status of nephron sparing surgery – open partial nephrectomy (OPN) for renal cell carcinoma in the minimal invasive era. The literature search was done using National Library of Medicine database (PubMed).

**Results:**

Early experience with laparoscopic partial nephrectomy is promising. It has an inherent advantage of less operative time, decreased operative blood loss and a shorter hospital stay at the expense of prolonged ischemia and operative time. Complex scenarios for partial nephrectomy such as centrally located tumor, tumor in a solitary kidney, predominantly cystic tumor, and multifocal disease probably are managed best with an open technique. All these challenging situations have been addressed successfully by experienced laparoscopic surgeons, therefore these conditions are best considered relative rather than absolute contraindications for laparoscopic partial nephrectomy.

**Conclusions:**

Laparoscopic partial nephrectomy faces the problem of technical complexity and availability of expertise. Open partial nephrectomy continues to be the gold standard for nephron sparing surgery.

## INTRODUCTION

Renal Cancer surgery has recently shown a trend towards parenchymal sparing and minimal invasive approach. The technique of nephron sparing surgery (NSS) has evolved through the phases of experimental surgery to surgery for patients with marginal renal reserve and now extending to those for elective setting.

There has been an increase in NSS done due to the dramatic increase in the lower stage lesions, better prognosis of incidentally diagnosed tumor[1,2] and the excellent outcome of NSS. The rationale for performing NSS is - increased longevity, improved health, early diagnosis in younger age group along with a better understanding and improvement in the surgical techniques. Moreover, rising incidence of incidentally diagnosed renal tumors[3,4] and up to 40% of benign renal masses in final histopathology have served as an impetus for the potential expansion of the indications for elective NSS. Where the selection of open NSS is a complex decision for physicians and patients alike, laparoscopic NSS is further associated with its technical difficulties.[5]

In this regard we summarize the current status of NSS – open partial nephrectomy (OPN) for renal cell carcinoma (RCC) in the era of minimal invasive surgery.

## NEPHRON SPARING SURGERY - OPEN PARTIAL NEPHRECTOMY

Radical nephrectomy has been considered as gold standard for the treatment of localized or locally advanced RCC. Laparoscopic radical nephrectomy has emerged as a less morbid alternative to open surgery in the management of low- to moderate-volume (8 to 10 cm or smaller), localized RCC cases with no local invasion, renal vein involvement, or lymphadenopathy. Nevertheless, studies have demonstrated the onco-surgical adequacy of NSS compared to the radical nephrectomy. Although operative time was found to be more with partial nephrectomy,[6] the perceived benefit of partial nephrectomy is preservation of renal parenchyma.

## EVOLUTION OF NSS

NSS started with cases for which the partial nephrectomy was a must (imperative) to those where it could be performed safely (elective). The current indication of NSS can appropriately be categorized into imperative, relative and elective [Table 1].

**Table 1:** Indications of nephron sparing surgery in practice[7]

Imperative	Relative–contralateral kidney is at threat	Elective
Solitary kidney	Local renal conditions – UPJO, NL, r.PN, VUR	Young healthy patient
Bilateral renal tumors	Systemic conditions – DM, HTN	RCC < 4 cm
Severe renal insufficiency	Genetic conditions – VHL, Papillary RCC	Exophytic tumor

UPJO: ureteropelvic junction obstruction, NL: nephrolithiasis, r.PN: recurrent pyelonephritis, VUR: vesico-ureteric reflux, DM: diabeties mellitus, HTN: hypertension, VHL: von hippal landau, papillary RCC: papillary renal cell carcinoma.

## EFFICACY OF NSS- OPN

Oncological Efficacy of OPN: Several authors have demonstrated that the oncological efficacy of NSS is equivalent to radical nephrectomy in small size and lower stage of RCC [Table 2].[8–12] Due to obvious reasons, cancer specific survival rates of partial nephrectomy depend on the indication, elective or absolute, for which the NSS is performed.

**Table 2:** Five year cancer specific survival in partial and radical nephrectomy.[7]

Study	No of patients		Follow-up (median, Months)	5-year cancer specific survival (%)	
		
	OPN	RN		OPN	RN
Lerner *et al.* 1996[9]	209	185	52	89	89
Belldegrun *et al.* 1999[10]	125	108	74	98	91
Lee *et al.* 2000[11]	183	79	40	95	95
Butler *et al.* 1995[12]	42	46	48	100	97

In an analysis of 909 cases of T1a tumors the disease specific survival and local tumor recurrence was 90-100%, 0-7.3% respectively.[13]

### Tumor Size – How Much is Too Much

Tumor size is the most important predictor of cancer related outcome.[1,8,9,14] This is an important selection criterion for partial nephrectomy. It was based on Cleveland Clinic data; the T1 stage was sub-divided into T1a and T1b. On retrospective review they showed that there was a significant fall in five and 10-year cancer specific survival rate and rise in the recurrence, if tumor size increases above 4 cm.[14] Fergany *et al.*[1] found cancer specific survival of 98% for tumor ≤ 4 cm size, of which 2% cases were elective [Table 3]. Tumor size, laterality and pathological stage were found to be significant risk factors for cancer specific death. Patients with tumors > 4 cm were significantly more likely to die of disease than those with tumors ≤ 4 cm (p=0.009). For each 1 cm increase in the tumor size the risk of death rose by 20%.[1]

**Table 3:** Disease-free survival in patients after nss evaluation by tumor size[7]

Study	Patients No. (<4 / 4-7cm)	Elective (%)	5-year cancer specific survival (%)	
			
			<4 cm	4-7cm
Lerner *et al.* 1996[9]	54	100	91	-
Belldegrun *et al.* 1999[10]	108(53/10)	58	100	90
Lee *et al.* 2000[11]	79	47	95	-
Butler *et al.* 1995[12]	46	13	100	-
Fergany *et al.* 2000[1]	107(43 / 21)	02	98	95
Hafez *et al.* 1999[14]	485 (240 / 80)	09	96	86

Subsequent studies have shown that in elective partial nephrectomy for T1b, in carefully selected patients, the oncological outcomes were equivalent to radical nephrectomy.[10,15–17] Belldegrun *et al.*[10] found equivalent disease free survival between partial nephrectomy for T1b and radical nephrectomy. Patard *et al.* did not find any significant difference for distant or local recurrence among patients undergoing radical nephrectomy or partial nephrectomy, in a multi-institutional trial, for T1b lesions.[15] In their analysis, Thresher *et al.* did not find any increase in cancer specific mortality between T1a and T1b lesions.[18] Although data for T1b NSS is encouraging, the careful selection of patients is of utmost important as the peripheral tumor location was more common in these series.

### Tumor Location

Previously, the proximity to the hilar vessel or centrally located tumor was considered to be contraindicated for NSS for two reasons:

- There is an increase in risk of local tumor recurrence due to difficulty in achieving the traditional 1 cm of margin of normal parenchyma. However, recent data suggested that margin size has no effect on local tumor recurrence as long as the final parenchyma margins are negative for tumor involvement.[19–21]
- The centrally located tumor is technically more challenging, as evident, by longer ischemia time and higher rate of pelvi-calycial system violation, associated morbidity.

Contrary to this, in a retrospective study, Hafeez *et al.*[22] showed that there was no significant difference between centrally and peripherally located tumors with respect to stage, grade, survival and tumor recurrence.

### Multi-focality

The associated multifocal tumor ranges from 4-25%.[23] The factors influencing the risk of multifocality are increasing tumor size (≥T2), papillary or mixed histopathology and vascular invasion.[24,25] Although the risk of multifocality with incidentally diagnosed small tumor is low, in a retrospective histopathological analysis, Schlichter *et al.*[26] found a mean distance of 26.4 mm among multifocal tumors. Therefore, tumor recurrence can be found not only from the tumor base but also from multifocal tumors. In this scenario the radical nephrectomy cases outweigh the partial nephrectomy as only ¼^th^ of these can be diagnosed preoperatively.[27]

### Symptomatic presentation

Renal cell carcinoma is characterized by diverse clinical manifestations. Small, localized tumors rarely produce symptoms and hence the diagnosis is often delayed. Symptomatic presentation is usually a sign of advanced disease. Studies have demonstrated that incidental neoplasm tends to be smaller, at a lower stage and grade and has better survival outcomes when compared with symptomatic RCC.[28] In a histopathological analysis, Gupta et.al. found that symptomatic RCCs had a higher nuclear grade and unfavorable histology specifically symptomatic T1b RCC, both of which are known to be associated with multicentricity and higher recurrence rate.[29] Renshaw *et al.* found that papillary RCCs were more aggressive as compared to clear cell RCC.[30] Kletscher *et al.* have shown that multifocality occurs at a significantly higher rate (P=0.011) with papillary and mixed histological pattern.[24] Licht *et al.* found that symptomatic renal tumors (>4 cm) treated with partial nephrectomy had a statistically significant worse prognosis. In their series five-year cancer-specific survival rates for incidental and symptomatic RCC were 94 and 83% respectively.[31] This prompted Patard *et al.* to propose a classification based on mode of presentation (incidental or symptomatic) combined with tumor size to stratify prognosis.[32] Similarly, Fergany *et al.* have shown a significant survival benefit not only for smaller lesions but also for those who had incidental presentation and with lower grade.[1] Although encouraging, prospective validation of the data in a large cohort is yet to be done.

### Surgical margin

Traditionally, at least 1 cm of normal renal parenchyma margin was considered a safe limit to reduce the risk of local tumor recurrence. However, after reviewing of data, Lau *et al.*[8] found that even when partial nephrectomy was performed with at least 3 mm of surrounding normal tissue margin with negative frozen section biopsy of tumor bed, the five-year recurrence free survival was 97%. Similarly, Sutherland *et al.*[21] analyzed data on 43 patients and showed that those with a mean tumor size of 3.2 cm and mean surgically resected margins of 2.5 mm, 41 (93%) had negative margins. Of the 41 with negative margins, 40 did not develop local tumor recurrence on a mean follow-up of 49 months. However, two of these with positive margins were radiological disease free at 39 and 62 months respectively.

### Metastatic relapse

Metastatic relapse of a tumor depends on the aggressive, biologic nature of the tumor. This is generally not recognized prior to the surgery; there are equal chances of relapse in partial or radical nephrectomy. Besides tumor stage, the other factors associated with metastatic relapse are tumor grade and histological subcategory of the tumor.

In Fuhrman's original report, the five-year survival rates for grades 1 to 4 were 64, 34, 31 and 10% respectively.[33] Nuclear grade proved to be the most significant prognostic factor for stage-I tumor in his series.[33] Castilla *et al.*[34] also reported a significant risk of disease progression with increasing Fuhrman nuclear grade (P<0.001). Studies have shown that with papillary and mixed histology RCCs, multi-focality occurs at a significantly higher rate (p=0.011) and is relatively more aggressive in terms of local tumor recurrence.[30,32]

## PRESERVATION OF RENAL PARENCHYMA AND RENAL FUNCTION

The main perceived benefit of OPN is preservation of renal parenchyma. Although the value of this preservation is well defined in imperative and relative scenarios, it remains unclear in pure elective cases. Studies have demonstrated a statistically significant decrease risk in chronic renal insufficiency among patients undergoing OPN, the clinical significance of these findings remains to be defined [Table 4].

**Table 4:** Comparison of preoperative and postoperative serum creatinine in radical nephrectomy (RN) and nephron sparing surgery (NSS)[7]

Study	No. of patients RN / NSS	Follow up (Months)	RN Preop / Postop	NSS Preop / Postop
Butler *et al.* 1995,[12]	41 /46	48	1.1 / 1.5	0.9 / 1.0
Indudhara *et al.* 1997,[37]	71 /35	41	1.0 / 1.9	0.9 / 0.8
Lau *et al.* 2000,[8]	164 / 164	47	1.1 / 1.4	1.1 / 1.2
McKiernan *et al.* 2002,[35]	173 / 117	26	1.0 / 1.5	1.0 / 1.0
Matin *et al.* 2002,[38]	35/82	1	1.0 / 1.4	0.9 / 1.0

Authors from Mayo clinic and MSKCC have compared radical nephrectomy and NSS for <4 cm tumor. They show that patients undergoing radical nephrectomy are more likely to have elevation of serum creatinine (>2.0mg/dl) and proteinuria.[8,35] The five-year survival rate was > 90% in both the series, independent of whether radical nephrectomy or NSS was performed. Though in the radical nephrectomy group there was no significant increase in requirement of dialysis, there was high risk of cardiovascular morbidity and mortality associated with chronic kidney disease that would have required dialysis.

Huang *et al.*[35] analyzed the MDRD-GFR (Chronic Kidney Disease was defined as GFR less than 60 ml/min/1.73 m^2^) on 662 patients. All those who underwent elective partial or radical nephrectomy for 4 cm sized tumors, a normal serum creatinine and two healthy kidneys were included. Prior to the surgery 26% of the patients had preexisting chronic kidney diseases despite having normal serum creatinine with normal appearing kidneys. After the surgery, the three-year freedom from GFR, which was less than 60 ml/min/1.73 m^2^ for NSS, was 80% compared to 35% after radical nephrectomy. Radical nephrectomy was found to be independent risk factor for the development of new onset chronic kidney disease in multi-variate analysis.

## RCC AND UNSUSPECTED MEDICAL RENAL DISEASE

On the basis of renal transplant literature it has been thought that radical nephrectomy will not cause serious long term side effects as long as the patient has a normal contralateral kidney despite the probability of a biochemical rise in serum creatinine because of the excision of the uninvolved renal parenchyma.

However, there is certainly a selection bias existing between renal donors and RCC patients. Donors are carefully selected and screened for medical diseases and are generally in the younger age group,[38,39] whereas the mean age of RCC is 61 years (sixth to seventh decade) when the associated co-morbidities are prevalent.[40]

As patient ages, particularly beyond 60 years, nephron atrophies and GFR progressively decrease.[41] Besides the age dependent nephron atrophy there is sub-clinical medical renal disease associated with RCC. During evaluation of non-neoplastic pathology in adjacent normal renal tissue in tumor nephrectomy specimen, Bijol *et al.*[42], found that 28% of specimens had vascular sclerotic changes, 62% had significant intrinsic abnormality, including diabetic nephropathy, glomerular hypertrophy, mesengial expansion and glomerulosclerosis. The sub-clinical medical renal disease associated with radical nephrectomy is a cause of concern for the worsening of overall renal function.

## INCREASED DETECTION OF BENIGN RENAL MASSES

An increment in detection of renal tumor at early stage, due to increased rate of radiological evaluation for non-specific complaints,[43,44] also increases the rate of detection of benign renal histology. The benign renal histology is found to be 20-40% of all small enhancing renal tumors in current literature.[45–48] NSS not only gives the chance to evaluate these lesions histopathologically, butalso avoids over treatment of these benign lesions.

## RISK OF DEVELOPING CARDIOVASCULAR DISEASE

With the emerging data, in 2003, the National Kidney Foundation, the American Heart Association and the Joint National Committee on Prevention, Detection, Evaluation and Treatment of High Blood Pressure categorized chronic kidney disease as independent risk factor for cardio-vascular disease and death[49–51] Subsequently, on analyzing data of 15,837 adults from 1988 to 1994 Foley *et al.* found increase in cardiovascular diseases from CKD I to V in those with cardiovascular risk factors. This ranges from 35% (stage-I and II), 84% (for stage-III), to 100% (for stage-V) for subjects who had two associated cardiovascular risk factors.[52]

## COST EFFECTIVENESS AND HOSPITAL STAY

Comparative studies between partial and radical nephrectomy have analyzed the operative time, blood loss, length of hospital stay and cost differences.[53,54] Uzzo *et al.*[53] did not find difference in hospital stay and cost between the groups. Shekarriz *et al.*[54] retrospectively compared 60 partial nephrectomy patients to 60 radical nephrectomy patients. Although the cost and complications were comparable, the mean operative time for partial nephrectomy was significantly longer (p<0.0001). No differences were found in the blood loss and transfusion rates between the groups.

## OPEN PARTIAL NEPHRECTOMY VS. LAPAROSCOPIC PARTIAL NEPHRECTOMY

In contemporary practice, OPN has become the gold standard for a single small renal tumor with demonstrated oncological and improved renal functional outcomes which are similar to those of radical nephrectomy.[1–3] Now the concept of nephron sparing surgery is further extended to minimal invasive approaches, namely, laparoscopic and probe ablative techniques. Among all, laparoscopic partial nephrectomy (LPN), duplicating the steps of open partial nephrectomy has grown rapidly. Studies have compared the outcome of LPN highlighting the potential risks and benefits associated with the approach.[55,56]

### Onco-surgical adequacy

#### Tumor location

The issues which make partial nephrectomy technically more demanding are achievement of negative margins, risk of local tumor recurrence, centrally located tumors and proximity to the hilar vessel as there are chances of prolonged ischemia time and violation of pelvi-calycial system. Recent data suggest that margin size has no effect on local tumor recurrence as long as the final parenchyma margins are negative for tumor involvement.[19–21] As far as OPN is concerned it has been found that there was no significant difference between centrally and peripherally located tumors with respect to stage, grade, survival and tumor recurrence.[22]

With the development of technique and expertise in laparoscopic partial nephrectomy for peripherally located small renal masses it has now been extended to more complicated centrally located renal tumors. In the past, LPN was advocated for non-renal hilar small tumors. However, in their series, Richstone *et al.* have demonstrated that those who had LPN for excision of a hilar tumor (renal tumor that came in direct contact with the renal artery and/or vein) did not develop any local recurrence or metastatic disease. The mean follow-up was 12.3 months (range 0.2 to 66), though long term results are still awaited.[57] Similarly, Desai *et al.*[58] demonstrated safety and efficacy of LPN for selected invasive renal tumors with intrarenal extension, even up to the collecting system. Intentional collecting system entry in such cases can be effectively repaired in a watertight manner by laparoscopic freehand suturing, albeit with longer mean operative and warm ischemia times, without adverse renal functional sequelae.[58] Though oncological results seem excellent, further follow-up is needed for accurate long-term assessment of this surgical approach. Therefore, resection of the renal sinus tumor is possible without adding to the risk of metastasis but increases the risk of surgical complications. Thus the successful LPN outcomes include selecting a tumor commensurate with the surgeon's laparoscopic experience.

#### Tumor size

Tumor size is the most important predictor of cancer related outcome.[1,8,9,14] This is an important selection criterion for partial nephrectomy. In their analysis, Fergany *et al.* found that patients with tumor > 4 cm were significantly more likely to die of disease than those with tumor ≤ 4 cm (p=0.009). For each 1 cm increase in the tumor size the risk of death rose by 20%.[1] However, Thresher *et al.* did not find increase cancer specific mortality on comparing T1a with T1b lesions.[18] The peripheral tumor location was more common in these series.

The size of renal lesions managed with laparoscopic partial nephrectomy has been increasing as there is better understanding of surgical technique, experience and increase in our surgical volume. But the perioperative and pathologic outcomes of LPN, when stratifying for size of renal lesion, is controversial. As the size of lesion increases there is increase in complexity in LPN and chances of positive surgical margins and local recurrence of the tumor. In a retrospective analysis Simon *et al.*[59] did not find any difference in positive tumor margin rate between T1a and T1b tumors for laparoscopic partial nephrectomy and suggested that LPN should be expanded to include patients with amenable > 4cm tumors. In a comparison of OPN and LPN, Permpongkosol *et al.*[60] also did not find significant differences for disease-free survival between the groups for T1 renal tumors. However, current reports do not convincingly favor LPN over OPN in terms of short or relative intermediate survival it may be due to technical complexity of the procedure. Therefore, OPN is the reference standard for NSS against which all minimally invasive NSS techniques should be measured.[61]

#### Multi-focality

As discussed earlier, the associated multifocal tumor ranges from 4-25%.[23] These tumors also have the propensity to escape detection on follow-up and there is always a fear that these tumors will become evident after the ‘intermediate follow-up’. The issue of LPN or OPN in a ‘nephron challenged’ patient is not under debate, there must be a caution in undertaking PN in patients based on tumor size alone. It is now recognized that the frequently cited multifocal lesions are no longer an argument against conservative surgery.[62] Thus, the role of partial nephrectomy, open or laparoscopic, depends on surgeon preference.

#### Surgical margin

It is now recognized that margin thickness has no real significance provided it is negative, even if excision is flush with the tumor capsule.[62] For surgical margins, intraoperative frozen section biopsies showed negative margins in most published series. Allaf *et al*.[63] found that final surgical margins were positive in ~2% of cases in their series. For recurrence rates in the short and intermediate follow-up, these range from 2.7[64] to 4.2%.[63] A recent study assessing the oncological outcomes of patients undergoing LPN for a renal tumor, and who had a positive surgical margin on final pathology, showed that a positive margin after LPN does not necessarily indicate residual disease.[66] The authors concluded that vigilant monitoring is mandatory and that while the mid-term outcomes are similar to those of patients with negative margins, a longer follow-up is necessary to determine the ultimate oncological outcome in this subgroup of patients.

#### Technical complexity

Operative time, Perioperative blood loss, Intraoperative Complications and Postoperative complications:

The complexity of intraperitoneal or retroperitoneal LPN has been increasing when attempting to reproduce the essential steps of OPN using contemporary laparoscopic instrumentation, namely early and complete vascular control, surface hypothermia, complete tumor excision, meticulous hemostasis, and precise reconstruction of the urinary collecting system and renal remnant[66] Despite advanced techniques that include the use of a harmonic scalpel and biological tissue adhesives such as fibrin glue[66,67] laparoscopic partial nephrectomy continues to result in prolonged operative time and an higher complication rate. Eng *et al*.[68] showed that operating time, need for collecting system repair and warm ischemia time was significantly more for lesions >2 cm. Other variables, namely rates of positive surgical margins, complications, estimated blood loss, conversion, and transfusion, were similar among the <2 and 2-4 cm groups.

### Ischemia time

Clamping the renal pedicle allows better vision for more accurate tumor excision with a safety margin and hemostatic suturing of the parenchymal defect. Clinical sequelae of warm ischaemic renal injury of approximately 30 minutes are minimal. Eng *et al*.[68] showed that operating time and warm ischemia time was significantly more for lesions >2 cm than <2cm tumors but the postoperative renal function did not differ among the groups with a short term follow-up. Desai *et al*.[69] had observed that advancing age and pre-existing azotaemia increase the risk of renal dysfunction after LPN, especially when the warm ischemia exceeds 30 minutes.

In their extensive study based on a multivariate analysis over 1800 patients retrospectively, Gill *et al*.[70] found that laparoscopic partial nephrectomy was associated with shorter operative time (*P*<0.0001), decreased operative blood loss (*P*<0.0001) and shorter hospital stay (*P*<0.0001)[Table 5].

**Table 5:** Comparison of characteristics: Laparoscopic Vs. Open partial nephrectomy[71]

Characteristics	Laparoscopic partial nephrectomy	Open partial nephrectomy	*P*-value
Preoperative characteristics			
Number of patients	771	1029	
ASA score ≥ 3 (%)	45.9	75.8	Ns
ECOG performance status ≥ 1 (%)	1.4	14.7	Ns
Symptomatic presentation (%)	8.8	33.5	Ns
Indications – Imperative/relative/elective (%)			
Clinical tumor size (range; cm)	0.5 – 7.0	0.6 – 7.0	Ns
Mean (cm)	2.7	3.5	
%≥4 cm	8.8	31.4	
Central tumors (%)	34.4	53.3	Ns
Preoperative S. creatinine (mean; mg/dl)	1.01	1.25	Ns
Peroperative characteristics			
Total operative time (mean; mins)	201	266	S
Warm ischemia time (mean; mins)	30.7	20.1	S
Estimated blood loss			
Mean (ml)	300	376	s
Transfusion (%)	4.5	5.1	ns
Postoperative characteristics			
Mean days hospital stay (range)	3.3 (1 – 42)	5.8 (1 – 96)	s
Pathological diagnosis (%)			
Benign	27.9	16.6	ns
Renal cell carcinoma	71.9	82.9	s
Others	0.26	0.49	ns
Postoperative S. creatinine (mean nadir; mg/dl)	1.18	1.42	ns
Post operative urological complications (%)	9.2	5.0	s
Post operative urine leak / hemorrhage (%)	3.1 / 4.2	2.3 / 1.6	ns / s
Post operative requiring subsequent procedure (%)	6.9	3.5	s

s: statistically significant; ns: statistically not significant.

The chance of intraoperative complications was comparable in the two groups. However, laparoscopic partial nephrectomy was associated with longer ischemia time (*P*<0.0001), more postoperative complications, particularly urological (*P*<0.0001), and an increased number of subsequent procedures (*P*<0.0001). Renal functional outcomes were similar at three months after laparoscopic and open partial nephrectomy with 97.9% and 99.6% of renal units retaining function, respectively. Three-year cancer specific survival for patients with a single cT1N0M0 renal cell carcinoma was 99.3% and 99.2% after laparoscopic and open partial nephrectomy, respectively.

### Other factors- hospital stay, patient convalescence and costs

Direct financial analysis demonstrated lower total hospital costs after LPN compared to OPN (4839 dollars+/− 1551 dollars versus 6297 dollars+/− 2972 dollars; *P* <0.05)[71] Laparoscopic partial nephrectomy can be cost equivalent to the open approach in managing small renal masses if the operating room time, length of stay, and equipment costs are closely monitored. The high cost of new technologies can be offset by shorter hospital stay and reducing operating time.[72]

## CONCLUSION

Early experience with laparoscopic partial nephrectomy is promising. It has inherent advantages of less operative time, decreased operative blood loss and a shorter hospital stay. However, continued efforts are required to develop laparoscopic renal hypothermia techniques and facilitate intra-renal suturing while minimizing the warm ischemia time. Although OPN remains the standard mode of NSS in patients who have localized RCC, overall data suggest that LPN, in the hands of an experienced laparoscopic surgeon, can be an effective treatment option in select patients with equivalent early cancer control. The increased warm ischemic times and postoperative urologic complications thus concern the careful selection of the patient. Complex scenarios for PN such as centrally located tumor, tumor in a solitary kidney, predominantly cystic tumor, and multifocal disease probably are managed best with an open technique. All these challenging situations have been addressed successfully by experienced laparoscopic surgeons, however, and these conditions are best considered relative rather than absolute contraindications to LPN.

Due to technical complexity and availability of expertise on laparoscopic partial nephrectomy there are difficulties in reproduction of results similar to select institutions with experience. Till then, open partial nephrectomy continues to be the gold standard for NSS and OPN is the reference standard for NSS in patients with a suspected renal malignancy against which all minimally invasive NSS techniques should be measured.
